# The Diagnosis and Treatment of Pseudoprogression, Radiation Necrosis and Brain Tumor Recurrence

**DOI:** 10.3390/ijms150711832

**Published:** 2014-07-03

**Authors:** Kashif Parvez, Aatif Parvez, Gelareh Zadeh

**Affiliations:** 1 Division of Neurosurgery, University Health Network, Toronto Western Hospital, University of Toronto, Toronto, ON M5T 2S8, Canada; E-Mail: kashif.parvez@utoronto.ca; 2 Department of Medical Imaging, Royal University Hospital, University of Saskatchewan, Saskatoon, SK S7N 0W8, Canada; E-Mail: aparvez@ucalgary.ca

**Keywords:** pseudoprogression, radiation necrosis, metastasis, glioma, recurrence

## Abstract

Radiation therapy is an important modality used in the treatment of patients with brain metastatic disease and malignant gliomas. Post-treatment surveillance often involves serial magnetic resonance imaging. A challenge faced by clinicians is in the diagnosis and management of a suspicious gadolinium-enhancing lesion found on imaging. The suspicious lesion may represent post-treatment radiation effects (PTRE) such as pseudoprogression, radiation necrosis or tumor recurrence. Significant progress has been made in diagnostic imaging modalities to assist in differentiating these entities. Surgical and medical interventions have also been developed to treat PTRE. In this review, we discuss the pathophysiology, clinical presentation, diagnostic imaging modalities and provide an algorithm for the management of pseudoprogression, radiation necrosis and tumor recurrence.

## 1. Introduction

Either diagnosis of brain metastatic disease or malignant glioma is a poor prognostic indicator in patients. Common metastatic tumors are lung cancer (48%), breast (15%), melanoma (9%) and colon cancer (5%) [1]. The global prevalence of brain metastases in cancer patients ranges from 8.5%–9.6% [2]. The incidence of new brain metastases in the USA is estimated at 7–14 persons per 100,000 [3]. Historically, patients with brain metastases were treated with corticosteroids, surgery and whole-brain radiation therapy (WBRT). Fractionated WBRT extended overall-survival from 3 to 5 months [4,5]. Surgical resection of a brain metastasis followed by WBRT increased overall-survival in patients as opposed to patients treated with biopsy and WBRT (40 *vs*. 15 weeks) [6,7]. With recent advances, patients can also be treated with stereotactic radiosurgery (SRS) and/or stereotactic radiotherapy (SRT). These treatments have increased the overall-survival and progression-free survival of brain metastasis patients [8,9].

The annual incidence of malignant gliomas is 5 per 100,000 [10,11]. In contrast to patients with secondary brain tumors, patients with malignant gliomas have a poorer prognosis with a median survival of 15 months [12]. Patients with a recurrence of a glioma have a median survival of 3–6 months [13]. Treatment of malignant gliomas involves surgical resection followed by external-beam radiation and temozolomide chemotherapy (TMZ) followed by six additional cycles of TMZ [14,15]. Radiation has been shown to improve survival by up to 12 months [15,16]. However, the improvement in overall-survival in both patients with brain metastatic disease and malignant gliomas is accompanied by complications such as post-radiation treatment effects (PTRE) including pseudoprogression and radiation necrosis.

Post-radiation treatment effects can be divided into pseudoprogression or radiation necrosis. Pseudoprogression often appears several weeks up to months (*i.e.*, 3+) after radiation treatment. The cause of pseudoprogression is the transient interruption of myelin synthesis secondary to radiation injury to oligodendrocytes. Many studies suggest a transient course with spontaneous recovery [17,18]. In contrast, radiation necrosis may appear months to several years after radiation therapy [19]. Radiation necrosis involves a space occupying necrotic lesion with mass effect and neurological dysfunction.

Magnetic resonance imaging (MRI) of the brain is performed to monitor patients after treatment. Patients may present with radiological findings suggestive of PTRE or tumor recurrence. It is challenging to differentiate post-radiation treatment effects from tumor recurrence by MRI alone. It is important to understand the radiological and clinical presentation that distinguishes these two entities in order to guide management.

In 1990, Macdonald *et al*., published criteria to evaluate the response to treatment of malignant gliomas [20]. This criteria used two-dimensional measurements of enhancing tumors from computer tomography (CT) scans, neurological status and corticosteroid use. The Response Assessment in Neuro-Oncology (RANO) Working Group published an updated set of criteria to include magnetic resonance imaging (MRI) [21]. The group defined treatment responses as complete response, partial response, stable disease and progressive disease. The criteria included T1 gadolinium enhancing disease, T2/FLAIR changes, new lesions, corticosteroid use, clinical status and the requirement for the response. The RANO criteria can be used to evaluate treatment response and tumor recurrence important to both clinical practice and for clinical trials. Clinicians face the challenge of diagnosing and managing patients that have a new or old lesion seen on follow up MRI, and determining whether the lesion is pseudoprogression, radiation necrosis or tumor recurrence. Here we review the literature and explore current developments to non-invasively diagnose tumor recurrence, pseudoprogression and radiation necrosis. The pathophysiology, clinical presentation, diagnostic imaging modalities and treatment of these entities are reviewed.

## 2. Methods

Four databases were searched since their inception. These included PubMed, EMBASE, MEDLINE and the Cochrane Library. Journal articles discussing either pseudoprogression, radiation necrosis or tumor recurrences in patients with brain metastatic disease or malignant gliomas were considered.

## 3. Pathophysiology

Pseudoprogression mimics tumor recurrence on MRI and has been reported in studies involving serial MRI up to 3 months post-treatment [22,23]. The rate of pseudoprogression seen has been upwards of 20% [24]. The T1 gadolinium enhancement in the resection cavity seen on MRI is transient and often resolves spontaneously without treatment. It is thought that the transient breakdown of the blood brain barrier explains the edema and contrast enhancement seen. In one study, patients who underwent re-resection, histologic analysis showed the absence of tumor recurrence and the effects of pseudoprogression are best described as radiation induced injury [25]. However, there have been no reports in tissue obtained from pseudoprogression to support the presence of the vascular changes, edema and fibrinoid exudate forming laminae as characteristically seen in radiation necrosis [26,27]. There is a suggestion that there is a correlation between *O*(6)-methylguanine-DNA methyltransferase (MGMT) methylation status and pseudoprogression with 91% of MGMT methylated tumors exhibiting pseudoprogression [28].

Overall, the pathophysiology of pseudoprogression and molecular changes associated with this phenomenon remains poorly understood. By comparison the mechanisms underlying radiation necrosis are better defined. The cells most sensitive to radiation are oligodendrocytes, endothelial cells and neural precursors. Apoptosis is the mechanism of cellular death by both p53 and p53-independent mechanisms [29,30]. Vascular injury initiates the process of necrosis. Histologic analyses have shown radiation necrosis occurs in the white matter and is associated with calcification, fibrinoid deposition, vascular hyalinization and endothelial thickening [31,32,33]. These changes result in chronic inflammation, chronic oxidative stress and inhibition of neurogenesis [34,35]. One key cytokine produced after irradiation is tumor necrosis factor-alpha (TNF-α). TNF-α upregulates other cytokines that lead to endothelial cell apoptosis, activated astrocytes and blood brain barrier permeability [36,37,38]. Vascular endothelial growth factor (VEGF) induced small vessel permeability and cause cerebral edema [39]. Following radiation therapy, increased VEGF expression has been found in the white matter. The degree of edema and breakdown of the blood brain barrier are correlated with the degree of VEGF expression [40]. The increase in expression of VEGF has been found to be associated with radiation necrosis (*i.e.*, late delayed reactions) and not pseudoprogression (*i.e*., early delayed reactions).

## 4. Clinical Presentation

The primary difference in clinical presentation between pseudoprogression and radiation necrosis is the time course. Pseudoprogression may occur weeks and up to 3 months after radiation treatment, whereas radiation necrosis presents 3 months to 3 years after treatment [22]. Pseudoprogression is commonly seen in asymptomatic patients with an enhancing lesion seen on follow up MRI. While the majority of patients with pseudoprogression remain asymptomatic, some patients present with complications due to the transient demyelination involved. These complications can include worsening of pre-existing symptoms, transient cognitive decline, subacute rhombencephalitis or somnolence syndrome [41].

Patients who develop radiation necrosis may either be symptomatic or asymptomatic with radiological abnormalities. They present with symptoms or signs of focal or diffuse necrosis that worsen neurological deficits [42]. Vascular injuries may manifest as Moya-Moya syndrome, stroke-like migraine attacks after radiation therapy (SMART syndrome), radiation induced aneurysms or radiation induced cavernous malformations or mineralizing microangiopathy [35,43]. Secondary or delayed cancers may also occur as well as tissue calcification, atrophy or leukoencephalomyelopathy [44,45]. Endocrine dysfunction producing hypogonadism or hypothyroidism have also been noted [41].

## 5. Diagnostic Imaging Modalities

Radiation therapy can have three outcomes depending on the time of occurrence and clinical presentation. Acute outcomes tend to occur early and generally at the time of radiation, whereas sub-acute or early-delayed outcomes tend to occur up to 3 months post therapy. Late-delayed outcomes (*i.e.*, radiation necrosis) generally occur anywhere from 3 months to years after therapy [24,46]. The incidence of radiation necrosis has been reported to range from 3%–24%, with a direct relationship to radiation dose, overall treatment duration, and irradiated brain volume [22,46]. A study by Ruben *et al*. postulated that other factors such as previous irradiation and even chemotherapy regiments likely increase the risk of radiation necrosis [47]. A phenomenon that can occur shortly after radiation therapy and often mimic tumor progression is the notion of pseudoprogression. Pseudoprogression represents an enlarging contrast enhancing lesion that stabilizes with time and is usually asymptomatic. The incidence of pseudoprogression has not been reported in a large series of studies, and there is a highly variable rate in the small case studies examined. Depending on the response criteria used and period examined, the reported incidence varies from 5.5% to 31% [24]. Different imaging techniques such as MRI, MRS, and PET have demonstrated promising results in an attempt to differentiate pseudoprogression from radiation necrosis and tumor recurrence. Unfortunately there is a lack of established criteria in the literature that can definitively characterize between these post treatment outcomes, and thus the current standard is follow-up examinations and histological analysis [48].

Several MR techniques have been implemented to aid in establishing the characterization of the status of post-treatment radiation effects, and include contrast administration, diffusion weighted imaging (DWI), diffusion tensor imaging (DTI), dynamic contrast enhancement (DCE-MRI), and magnetic resonance spectroscopy (MRS). Diffusion weighted imaging is based upon water molecule motion, and can provide information about the structural integrity of brain tissue. The apparent diffusion coefficient (ADC) value has been noted in several studies to be higher in radiation necrosis than recurrence; however, the values reported are inconsistent and likely represent technical factors [46,49]. The principle of DTI is similar to DWI in that it measures the directionality of proton movement, which can be used to compute maps of fractional anisotropy, mean diffusivity, and ADC. Fractional anisotropy has been reported to be lower in tumor than white matter tracts, and even lower in radiation necrosis, likely secondary to the destruction of normal axonal fibers. A study by Xu *et al*. utilized DTI and suggested that the mean ADC ratios for radiation necrosis were 1.62 and 1.34 in recurrent tumor [50]. Another method used for DTI is that when diffusion restriction is seen on ADC images, it favors recurrent tumor *vs.* radiation necrosis, which does not demonstrate diffusion restriction.

DCE-MRI perfusion has been studied recently which measures cerebral blood flow and volume, and can be accurately measured in cases of blood-brain barrier disruption. In a study by Larsen *et al*., measurements of the cerebral blood volume (CBV) were performed on patients with contrast enhancing lesions on MRI, which correlated well with FDG-PET examinations [51]. The lesions that regressed demonstrated a low CBV (less than 1.7 mL/100 g) and generally corresponded to a region of metabolic inactivity on FDG-PET, representing radiation necrosis. Lesions that progressed demonstrated a high CBV (greater than 2.2 mL/100 g) generally corresponded to a region of metabolic activity on FDG-PET, representing tumor recurrence. Thus the conclusion of the study by Larsen *et al*. was that an absolute CBV threshold of 2.0 mL/100 g could detect the regression or progression of a lesion, with a reported sensitivity and specificity of 100%.

Another MR technique that can be very useful is MRS, which measures the relative composition of specific metabolites in a single or multiple voxels. The most common metabolites in MRS are *N*-acetylaspartate (NAA), choline, creatine, lipid and lactate, all of which will have differing levels and ratios based on the underlying mechanism of injury. Several studies have demonstrated that tumor recurrence had higher Cho/Cr and Cho/NAA values than those with radiation necrosis [46,52,53]. In a study by Kamada *et al*., it was shown that radiation necrosis generally had increased lactate/Cr ratios, and decreased Cho/Cr ratios.

The concept of a lesion quotient (LQ), which is defined as the area of a hypointense nodule on a T2 weighed MR image divided by its area on a contrast-enhanced T1 weighted image, shows promising results. A study by Dequesada *et al*. used the LQ to help distinguish recurrent metastasis from radiation necrosis due to radiosurgery. Their study determined that a LQ greater than 0.6 was seen in recurrent tumor (100% sensitivity, 32% specificity), less than 0.3 in radiation necrosis (80% sensitivity, 96% specificity), and greater than 0.3 in combined disease (15% sensitivity, 100% specificity) [54]. An attempt to validate these results was performed by Stockham *et al*., where 51 patients underwent surgical intervention with histopathological confirmation. In their study, the sensitivity and specificity for recurrent tumor with a LQ > 0.6 was 59% and 41% respectively. The sensitivity and specificity for radiation necrosis with a LQ < 0.3 was 8% and 91% respectively. The sensitivity and specificity for a combination of recurrent tumor and radiation necrosis was 0% and 64% respectively. The two studies differed dramatically with respect to the ability of LQ to help differentiate post treatment radiation effects and, as mentioned in the study by Stockham *et al*., may be due to different time periods, changes in practice, patient demographics and technological differences [55]. Thus, while the ability to calculate the lesion quotient can be another important tool in the differentiation of tumor recurrence *vs.* radiation necrosis, it still requires further validation in the literature.

While the lesion quotient is an objective tool utilizing T2 and T1 post contrast imaging, “T1/T2 mismatch” is a more subjective tool that was defined by Kano *et al*. as the enhancement on T1-weighted MRI scans that did not match any corresponding and similar low-intensity mass on T2-weighted images [56]. They also defined “T1/T2 match” as the border of a nodule or lesion wall on the T2-weighted MRI scans that matched with the border on the T1-weighted enhanced images (including partially matched border on both images). Kano *et al*. examined 68 patients and found that lesions with “T1/T2 match” were highly correlated with tumor progression (*p* < 0.0001) and lesions with “T1/T2 mismatch” were highly correlated with radiation necrosis (*p* < 0.0001). The sensitivity and specificity of T1/T2 mismatch was reported to be 83.3% and 91.1% respectively. An attempt to validate the results by Kano *et al*. was performed by Leeman *et al*. where 49 patients with brain metastases were analyzed [57]. The results were discordant, demonstrating a lack of any significant correlation between T1-weighted enhancing images with T2-weighted images (*p* = 0.720 for univariate and 0.489 for multivariate analysis of pre-operative T1/T2 mismatch).

Positron Emission Tomography (PET) utilizes the ability of brain tissue to uptake radiotracer (most commonly fludeoxyglucose; a glucose analog) and demonstrates metabolically active lesions on cross-sectional imaging, such as CT or MRI. Tumor recurrence will usually appear as metabolically active lesions, while radiation necrosis will appear metabolically inactive. However there are limitations that can arise during interpretation of FDG-PET, such as differentiation from normal cortical uptake, and hence why amino acid analogs such as F-DOPA and C-MET may be more suitable for tumor recurrence [58,59]. SPECT is another nuclear medicine examination that utilizes radiotracer uptake, such as Thallium-201. Normal brain tissue does not typically demonstrate Th-201 uptake, and thus is reported to have a high sensitivity and specificity for tumor recurrence [60]. A summary of the varying characteristics for the different imaging techniques is presented in Table 1.

**Table 1:** Differentiating between tumor recurrence and radiation necrosis using different diagnostic imaging modalities.

Imaging Modality	Tumor Recurrence	Radiation Necrosis
DWI/DTI	High FA	Low FA
	Diffusion restriction	No diffusion restriction
	Lower ADC values	Higher ADC values
DCE-MRI	Elevated CBV > 2.0 mL/100 g	Decreased CBV < 2.0 mL/100 g
MRS	Higher Cho/Cr and Cho/NAA	Increased lactate/Cr and decreased Cho/Cr
LQ	>0.6	<0.3
PET/SPECT	metabolic activity/increased radiotracer uptake	no metabolic activity/no radiotracer uptake

Significant improvements have been made for a non-invasive approach to post-treatment outcomes, yet a major diagnostic dilemma that remains is the ability to differentiate between radiation necrosis, pseudoprogression and tumor recurrence. While contrast enhanced MRI has its benefits, a multi-modality approach has become crucially important and newer techniques show very promising results.

## 6. Management Considerations

### 6.1. Corticosteroids

Often patients who present with either pseudoprogression or radiation necrosis may be asymptomatic and only require close surveillance with serial imaging. Depending on the size, edema, location and mass effect of the lesion, intervention may be indicated in symptomatic patients. In patients with pseudoprogression that have worsening of their symptoms due to transient cerebral edema, a short course of corticosteroids is warranted. Corticosteroids inhibit the pro-inflammatory response associated with the transient demyelination seen in pseudoprogression. Agents such as dexamethasone, reduce the radiation induced cytokine response, reduce the leakiness of the blood brain barrier and thus reduce the degree of edema [61]. Corticosteroid use for long durations can give rise to side effects such as steroid myopathy, osteopenia, glucose intolerance and Cushing’s syndrome [44].

### 6.2. Bevacizumab

Given the role of VEGF in causing small vessel permeability and breakdown of the blood brain barrier after radiation injury, several studies have shown bevacizumab may be an effective therapy. Bevacizumab is an anti-VEGFA monoclonal antibody commercially available as Avastin (Genentech, South San Francisco, CA, USA). A randomized double blind placebo controlled trial showed a benefit to treating radiation necrosis in symptomatic patients with bevacizumab [62]. There is an improvement in the contrast enhancing volumes and FLAIR MR sequences in patients treated with bevacizumab [63]. Patients treated with bevacizumab can be maintained on a lower dosage of corticosteroids [64]. Re-challenging patients with bevacizumab is effective in treating recurrent radiation necrosis after bevacizumab treatment [65].

### 6.3. Anticoagulation

The histologic characteristics of the necrotic lesion include abnormal vasculature, thrombosis, fibrinous exudates, telangiectasias, dilated and tortuous vessels with thickened and enlarged endothelial cells. The features lead to disruption of the blood brain barrier and anticoagulants are hypothesized to dissipate these features [66]. Heparin and warfarin inhibit cytokine release, prevent coagulation and inhibit platelet aggregation [67]. The possibility that anticoagulants could be used to treat radiation necrosis was noted in two patients who improved after being treated for deep-vein thrombosis [68]. Another study used heparin and warfarin to treat eight patients with radiation necrosis [67]. Patients with radiation necrosis were noted to improve within days of initiation of heparin therapy and within 4 weeks if treated with warfarin. These studies contained small sample sizes and lacked the statistical power to determine a rate of hemorrhagic complications.

### 6.4. Hyperbaric Oxygen Treatment

Hyperbaric oxygen treatment (HBOT) has been studied as a treatment for radiation necrosis. To perform HBOT, patients are placed in a chamber with 100% oxygen at 2.5 times atmospheric pressure. This is performed up to 5 times a week for up to 30–40 treatments. The hypothesis is that increasing brain parenchymal oxygen concentration stimulates angiogenesis and restores the blood supply affected by radiation induced vascular injury. There is no data from a randomized control trial to suggest that HBOT can be used to treat radiation necrosis and only case reports that provide any evidence [69,70]. A reduction in radiation necrosis from 20% to 11% of patients has been in seen in one study when HBOT was used prophylactically. Patients were treated with a HBOT regimen beginning one week after stereotactic radiosurgery. The regimen included 15 min of compression with air then an hour of 100% oxygen inhalation at 2.5 times atmosphere followed by 10 min of decompression for 20 sessions [71]. HBOT may be an option as a prophylactic treatment in patients with a high likelihood of developing radiation necrosis.

### 6.5. Surgery

Surgical resection is an option in symptomatic patients with radiation necrosis that present with lesions in surgically accessible areas. Surgery reduces the mass effect, reduces edema, lowers intracranial pressure and provides confirmatory tissue diagnosis. Intraoperatively, radiation necrosis and tumor recurrence can be differentiated. Radiation necrosis appears avascular, firm and pasty while recurrent tumors often appear vascular, soft and purple [72]. Post-operatively, the majority of patients experience lasting clinical improvement [73].

### 6.6. Laser Interstitial Thermal Therapy

In the peri-necrotic core, dysfunctional astrocytes, microglial cells and oligodendrocytes produce vasoactive factors (*i.e.*, VEGF) that stimulate cytokine release and increase the permeability of the blood brain barrier. Laser interstitial thermal therapy (LITT) involves focusing a laser probe within the necrotic core and creating temperatures of 45–55 °C. Thermal coagulation destroys the peri-necrotic region of abnormal angiogenesis. LITT is hypothesized to remove VEGF from the peri-necrotic region and create a zone of inactive, VEGF-depleted coagulative necrosis. The coagulative necrosis is then resorbed [74]. This therapy is currently being investigated in a phase 2 study, Laser Ablation After Stereotactic Radiosurgery.

### 6.7. Oral Vitamin E administration

The combination of pentoxifylline and oral Vitamin E therapy have been used to treat radiation induced effects in skin cancer patients [75]. A pilot study by Williamson *et al*. showed a decrease in cerebral edema in patients treated with a combination of pentoxifylline and oral Vitamin E after stereotactic radiosurgery [76]. Vitamin E scavenges reactive oxygen species generated from the oxidative stress triggered by radiation. This protects cell membranes from lipid peroxidation [75]. It is postulated that Vitamin E promotes normal connective tissue repair without scarring after radiation [77,78]. A phase II clinical trial is currently underway examining the use of Trental and Vitamin E for the prophylaxis of radiation necrosis.

### 6.8. Algorithm for Diagnosis and Management of Patients

**Figure 1:**
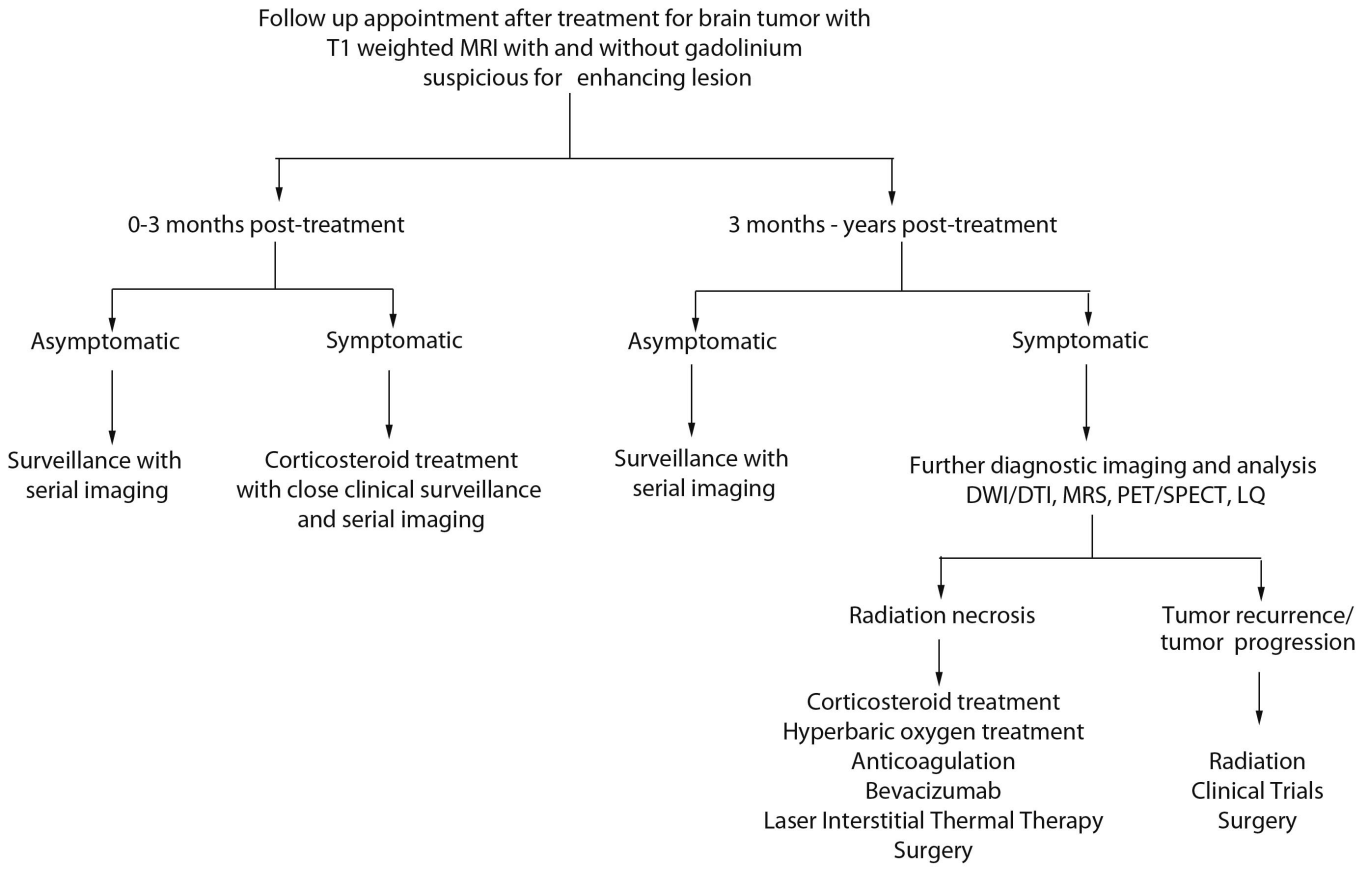
An algorithm to aid in the diagnosis and management of patients with pseudoprogression, radiation necrosis and tumor recurrence/progression.

In Figure 1, we provide an algorithm to aid in the diagnosis and management of patients that present to the clinic after treatment for their brain tumor with a suspicious enhancing lesion on T1-weighted MRI. If the time course has been 0–3 months post-treatment, an asymptomatic patient may be followed with serial imaging. If the patient is symptomatic, they may be experiencing pseudoprogression and may require corticosteroid treatment with closer clinical surveillance and serial imaging. If the time course been greater than 3 months but the patient remains asymptomatic, we recommend clinical surveillance with serial imaging. In symptomatic patients and if diagnostic imaging resources are available, further diagnostic imaging modalities can be used to aid in the distinction between radiation necrosis and tumor recurrence/progression. In the event that several diagnostic imaging modalities suggest that tumor recurrence or tumor progression has occurred, then management considerations can include radiation therapy, surgery or clinical trials. If the diagnostic imaging suggests that a symptomatic patient has radiation necrosis, a whole host of treatments can be explored including corticosteroid treatment, HBOT, bevacizumab, LITT or surgery.

## 7. Conclusions

Radiation therapy is currently a mainstay of treatment for brain metastases with proven efficacy for local control of disease. However as patient outcomes and overall survival improves, so does the paradoxical increase in presentation of many post-radiation effects principally in the form of pseudoprogression, or radiation necrosis. The pathophysiology of these entities is poorly understood and consequently their management remains largely symptomatic control at this stage.

One of the main challenges for the treating clinician is how best to diagnose and manage symptomatic patients with MRI suggestive of either tumor recurrence or radiation necrosis. With diagnostic imaging modalities (*i.e*., DWI/DTI, MRS and PET/SPECT) it is still challenging to differentiate between radiation necrosis and tumor recurrence. At this stage there is no single diagnostic imaging modality that allows for the differentiation between the two. There are varying levels of sensitivity and specificity associated with each modality and a combination of modalities can be utilized. A better understanding of the biology and molecular changes that follow radiation therapy, both acute and long-term, is important to allow establishing non-invasive imaging biomarkers that can distinguish radiation related changes and ultimately effective therapeutic strategies.
